# Cone-Beam Computed Tomography Assessment of Quality of Endodontic Treatment and Prevalence of Procedural Errors in Mandibular Molars

**DOI:** 10.1155/2023/3558974

**Published:** 2023-05-19

**Authors:** Ahmad Nouroloyouni, Amin Salem Milani, Ata Etminan, Sara Noorolouny, Elham Tavakkol, Hesam Mikaieli Xiavi, Nazila Ghoreishi Amin

**Affiliations:** ^1^Department of Endodontics, School of Dentistry, Ardabil University of Medical Science, Ardabil 5618985991, Iran; ^2^Department of Endodontics, School of Dentistry, Tabriz University of Medical Science, Tabriz 5165665931, Iran; ^3^Department of Pediatric Dentistry, Ardabil University of Medical Science, Ardabil 5618985991, Iran; ^4^Department of Radiology, University of California, Los Angeles, USA; ^5^Department of Radiology, Ardabil University of Medical Science, Ardabil 5618985991, Iran; ^6^Department of Radiology, University of Southern California, Los Angeles, USA

## Abstract

**Objectives:**

This study assessed the quality of endodontic treatment and the prevalence of procedural errors in permanent mandibular molars using cone-beam computed tomography (CBCT).

**Materials and Methods:**

This cross-sectional study was conducted on 328 CBCT scans (182 females and 146 males) of endodontically treated mandibular molars retrieved from the archives of two radiology centers in Ardabil city, Iran, in 2019. Mandibular molars were evaluated on sagittal, coronal, and axial sections regarding obturation length, obturation density (voids), missed canals, broken instruments, apical perforation, strip perforation, ledge formation, transportation, root fracture, root resorption, and periapical lesions by a senior dental student under the supervision of an oral and maxillofacial radiologist and an endodontist. Differences between the frequency of procedural errors and tooth type and gender were analyzed by the chi-square test.

**Results:**

The frequency of underfilling, missed canals, overfilling, voids, apical perforation, transportation, ledge formation, broken instruments, root fracture, strip perforation, root resorption, and periapical lesions was 34.8%, 17.4%, 16.8%, 14.3%, 7.3%, 6.1%, 4.3%, 3%, 1.2%, 0.6%, 5.5%, and 46%, respectively. The frequency of root fracture was significantly higher in females than in males (*P* < 0.05). The prevalence of underfilling was the highest in right second molars (47.2%), followed by right first molars, left second molars, and left first molars (*P* < 0.005). The frequency of transportation was maximum in right first molars (10%), followed by right second molars, left first molars, and left second molars (*P* < 0.04).

**Conclusion:**

Underfilling, missed canals, and overfilling were the most prevalent procedural errors in mandibular molars in our study population.

## 1. Introduction

Eradication of bacteria from the root canal system is the key to a successful endodontic treatment [1]. Adequate elimination of bacteria and prevention of their recolonization can increase the success of endodontic treatment to 94% [2, 3]. A successful endodontic treatment requires optimal cleaning and shaping of the root canal to the working length while maintaining the original canal path, followed by complete obturation of the root canal system and the creation of a hermetic seal.

The success rate of endodontic treatments performed by general dentists is reportedly 60% to 75% [4]. Procedural errors are among the main factors responsible for the suboptimal success rate of endodontic treatments performed by general dentists [5, 6]. Procedural errors can adversely affect the treatment prognosis and may not be easily corrected. Some procedural errors may require endodontic re-treatment, apicoectomy, or even tooth extraction. Root canal transportation, apical perforation, strip perforation, access cavity perforation, and instrument fracture are among the commonly occurring procedural errors [7]. Obturation errors such as underfilling and overfilling, sealer extrusion and void formation are also common [8]. Working length significantly affects the treatment outcome, such that root fillings shorter than the apex by 2 mm decrease the success rate to 68% to 77%, and overfilled canals exceeding the apex have a success rate of approximately 75% [9, 10]. Root perforation causes infection of the periodontal ligament and alveolar bone and can lead to endodontic treatment failure [11]. Also, a significant correlation exists between instrument fracture in the canal and poor treatment outcome [12].

Endodontic treatment of molar teeth is more challenging, and requires more caution compared with anterior and premolar teeth, and is associated with a higher rate of procedural errors. The reason is their difficult accessibility and anatomical complexities [13]. Mandibular molar teeth have the highest rate of ledge formation [14].

The quality of root canal treatment is routinely assessed by periapical radiography [15, 16]. However, periapical radiography provides a 2D image of 3D root canal anatomy, and cannot reveal the details of endodontic treatment. CBCT has been designed to generate accurate three-dimensional images of teeth and their adjacent tissues. This is typically accomplished with a significantly lower effective dosage than traditional medical computed tomography [17]. Cone-beam computed tomography (CBCT) enables 3D assessment of the quality of root fillings (voids, underfilling, overfilling, and extrusion of sealers) [8]. It is also valuable for the assessment of the complex morphology of molar teeth and can greatly help in cases where the conventional modalities fall short [18–20]. Periapical disease may be detected sooner using CBCT compared with periapical views, and the true size, extent, nature, and position of periapical and resorptive lesions can be assessed [21]. The CBCT is also a highly advantageous educational instrument. With the aid of CBCT, the quality of endodontic treatments and iatrogenic errors can be determined utilizing existing archives that reveal the weak points of clinicians, allowing for target-based learning to reduce these weaknesses [22].

In general, root canal therapy includes four steps: diagnosis, access cavity preparation, cleaning and shaping, and obturation. The success of each step depends on the correct implementation of the previous step. Mastering all four steps and having adequate knowledge about the possible procedural errors that may occur in each step can improve the overall quality of the procedure. Accordingly, knowledge about the most common procedural errors is imperative to prevent their occurrence [23, 24]. This study aimed to assess the quality of endodontic treatment and the prevalence of procedural errors in permanent mandibular molars using CBCT.

## 2. Materials and Methods

This cross-sectional study evaluated 328 CBCT scans (182 females and146 males) of endodontically treated mandibular molars (*n* = 328) retrieved from the archives of two radiology centers in Ardabil city, Iran, in 2019. The CBCT scans had been taken for diagnostic and treatment planning purposes not related to this study. The sample size was calculated according to Morgan's table. The study was approved by the ethics committee of Ardabil University of Medical Sciences (IR.ARUMS.REC.1398.155). The patients consented to the use of their CBCT scans for research purposes at the time of radiography.

The inclusion criteria were high-quality CBCT scans visualizing endodontically treated mandibular molars. Mandibular molars with prosthetic crowns, intracanal posts, and deep restorations were excluded due to artifact generation. The CBCT scans were selected by convenience sampling.

A trained and calibrated senior dental student evaluated the endodontically treated mandibular molars on CBCT scans under the supervision of an oral and maxillofacial radiologist and an endodontist. Mandibular molars were evaluated in the sagittal, coronal, and axial planes regarding the length of root filling, density of root filling (presence of voids), number of missed canals, presence of broken instruments, apical perforation, strip perforation, ledge formation, transportation, root fracture, root resorption, and presence of periapical lesions.

To ensure the validity of assessments, 25% of the CBCT scans were randomly selected and re-evaluated by the supervising oral and maxillofacial radiologist and endodontist. Also, to assess intraexaminer reliability, all CBCT scans were evaluated by the senior dental student 10 days after their primary assessment, and the agreement between the findings in the first and second observations was calculated using Cohen's Kappa, yielding perfect agreement (Kappa = 1).

SPSS version 25 was used to analyze the data. The frequency and percentage of procedural errors (underfilling, overfilling, obturation density, missed canals, broken instruments, apical perforation, strip perforation, ledge formation, transportation, root fracture, root resorption, and periapical lesions) were calculated and reported in general and separately for different canals of mandibular right and left first and second molars. The frequency distribution of procedural errors based on gender and tooth type (right/left first/second molars) was analyzed by the chi-square test at the 0.05 level of significance.

## 3. Results

Totally, 328 CBCT scans of 182 females (55.5%) and 146 males (44.5%) were evaluated. Of 328 endodontically treated mandibular molars, 90 (27.4%) were right first molars, 80 (24.4%) were left first molars, 72 (22%) were right second molars, and 86 (26.2%) were left second molars.

Of all teeth, 2 (0.6%) had one single canal, 31 (9.5%) had two canals, 235 (71.6%) had three canals, 56 (17.1%) had four canals, and 4 (1.2%) had five canals.

Of all patients, the age of 13 had not been disclosed in their records. The mean age of the remaining 315 patients was 41.22 ± 11.84 years (range 15 to 74 years).

The frequency of underfilling, missed canals, overfilling, voids, apical perforation, transportation, ledge formation, broken instruments, root fracture, strip perforation, root resorption, and periapical lesions was 34.8%, 17.4%, 16.8%, 14.3%, 7.3%, 6.1%, 4.3%, 3%, 1.2%, 0.6%, 5.5%, and 46%, respectively.

Table 1 presents the frequency of underfilling, overfilling, and voids in different canals of endodontically treated mandibular molars. As shown, underfilling had the highest frequency in the mesiobuccal canals (68.4%), followed by the mesiolingual canals (64.9%). Overfilling had the highest frequency in the distal canals (56.4%), followed by the mesiolingual canals (40%). The highest frequency of voids was noted in the mesiolingual canals (57.4%), followed by the mesiobuccal canals (42.6%).

Table 2 presents the frequency of missed canals, ledge formation, and apical perforation in different canals of endodontically treated mandibular molars. The distolingual canal (40.4%) was the most commonly missed canal, followed by the mesiobuccal canal (24.6%). Ledge formation had the highest frequency in mesiolingual canals (57.1%), followed by mesiobuccal canals (42.9%). Apical perforation had its maximum frequency in the distal canals (58.3%), followed by the mesiobuccal canals (33.3%).

Table 3 indicates the frequency of canal transportation, root resorption, and apical lesions in different canals of endodontically treated mandibular molars. As shown, the distal canals had the highest frequency of canal transportation (50%), followed by the mesiobuccal canals (40%). Root resorption had maximum frequency in the distal canals (61.1%), followed by the mesial canals (55.6%). Apical lesions had the highest frequency in the mesial canals (82.1%), followed by the distal canals (56.3%).

Table 4 compares the frequency of procedural errors in males and females. The chi-square test showed that the frequency of root fracture was higher in females than males (*P*=0.025). No other significant differences were noted (*P* > 0.05).

Table 5 compares the frequency of procedural errors in the right and left mandibular first and second molars. A significant difference was noted in the frequency of underfilling among different tooth types (*P* < 0.005) such that its frequency was maximum in right second molars (47.2%) and minimum in left first molars (20%). The frequency of missed canals was also significantly different among different teeth (*P*=0.010) such that right second molars had the highest frequency of missed canals (30.6%), while left first molars had the lowest frequency of missed canals (12.5%). The frequency of canal transportation was also significantly different among different teeth (*P*=0.037) such that its frequency was maximum in right first molars (10%) and minimum in left second molars (1.2%). No other significant differences were found (*P* > 0.05).

Table 6 shows the frequency of root canal morphology of mandibular molars, according to the Vertucci's classification. As shown, the most common root morphology was type IV and II in mesial root of first molars, I and II in distal root of first molars, IV and II in the mesial root of second molars, and I in distal root of second molars.

## 4. Discussion

This study assessed the quality of endodontic treatment and the prevalence of procedural errors in permanent mandibular molars using CBCT. CBCT has been designed to generate accurate three-dimensional images of teeth and their adjacent tissues. This is typically accomplished with a significantly lower effective dosage than traditional medical computed tomography [17]. In response to the rising demand for safer, more predictable treatments, numerous professionals have adopted CBCT to enhance visualization and comprehension in complex clinical settings [25, 26]. By reconstructing images in 3D, CBCT can aid in the detailed evaluation of structures' anatomy and morphology. CBCT images always aid in locating a larger number of roots or canals than conventional techniques [27]. Notably, mandibular molars exhibit a high degree of variety in canal configurations (Table 6).

CBCT is also helpful prior to periapical surgery; the size of the cortical and the relationship of anatomical structures like the maxillary sinus and inferior dental nerve to the root apices shall be evaluated using CBCT scans [17]. CBCT images are useful for analyzing treatment outcomes too. CBCT can detect periapical disease earlier than periapical views, and the true size, extent, character, and position of periapical and resorptive lesions can be evaluated [21]. The CBCT is also a highly advantageous educational instrument. With the aid of CBCT, the quality of endodontic treatments and iatrogenic errors can be determined utilizing existing archives that reveal the weak points of clinicians, allowing for target-based learning to reduce these weaknesses [22].

Hendi et al. [28] evaluated the procedural errors in endodontic treatments performed by dental students in Hamadan, Iran, and reported that apical transportation, ledge formation, and apical perforation were more common in molar teeth. Also, AlRahabi [29] reported the maximum frequency of errors in mandibular molars (43.1%). The higher frequency of errors in molar teeth is due to their difficult accessibility and anatomical complexities. Accordingly, mandibular molars were evaluated in this study.

The present results revealed that the prevalence of periapical lesions was 46%. Such a high rate is probably related to poor quality of chemomechanical preparation of the root canal system or poor quality of obturation. Tavares et al. [30] reported that although the quality of the coronal restoration has an influence on the treatment outcome, the quality of the endodontic treatment was the most important factor for the success of root canal treatment and the absence of a periapical lesion. AlRahabi [29] evaluated the technical quality of endodontic treatments and iatrogenic errors by undergraduate dental students in a dental school in Saudi Arabia and reported optimal technical quality in 68.9% of the teeth. Eskandarloo et al. [31] evaluated the technical quality of endodontic treatments performed by 5th year dental students by radiography in Hamadan, Iran, and reported that the technical quality of root fillings was acceptable in only 10.4% of the cases. Awooda et al. [32] radiographically evaluated the technical quality of endodontic treatments performed by dental students in Sudan and reported that the overall quality was optimal in 55.5% of the cases. In the current study, the incidences of periapical lesions was marginally higher in males and on the right side of the mandible, but these differences were not statistically significant. Alghamdi and Almehmudi [33] also reported that the prevalence of periapical lesion was significantly associated with male gender and the right mandibular side, comparable to our findings.

In the present study, underfilling (34.8%) was the most frequent procedural error, followed by missed canals (17.4%), overfilling (16.8%), and voids (14.3%). The results of previous studies have been variable regarding the most frequent procedural errors. In the study by AlRahabi [29], the frequency of underfilling and overfilling was 49.9% and 24.1%, respectively. These values were 17.1% and 12%, respectively, in the study by Eskandarloo et al. [31], 17.8% and 10.2%, respectively, in a study by Ehsani et al. [34] on the quality of endodontic treatments performed by undergraduate dental students in Babol, Iran, and 34.5% and 4.2%, respectively, in a study by Barrieshi-Nusair et al. [35] on dental students in Jordan. The frequency of overfilling was 18.2% by undergraduate dental students in a study by Haji-Hasani et al. [36] in Qazvin, Iran. The frequency of underfilling and overfilling was 37.45% and 6.25%, respectively, in a study by Jamani and Fayyad [37] in Jordan. These rates were 23.3% and 15.3%, respectively, in a study by Mozayeni et al. [38] in a dental school in Tehran, Iran, 21% and 9%, respectively, in a study by Lynch and Burke [39], and 37.3% and 7.8%, respectively, in a study by Ilgüy et al. [5] on dental students in Turkey. Nouroloyouni et al. [22] also reported that underfilling was the most common error in the second and first mandibular premolars (9.5% compared with 9.2%), respectively. In addition, overfilling was the second most common error in this study (6.3%). Another study reported the frequency of underfilled and overfilled root fillings to be 10.5% and 5.42%, respectively [40]. Variations in the reported values can be attributed to different methodologies, definitions (e.g. the acceptable length of root fillings), instrumentation and obturation techniques, experience and expertise of clinicians, patient cooperation, quality of assessment, type and quality of radiographs used to judge the quality of treatments, different quality of instructions, and differences in sample size and tooth type. Moreover, it should be noted that underfilling and overfilling are often secondary to incomplete or incorrect implementation of previous steps.

The present results showed that the frequency of voids was 14.3%. This value was 12.6% in the study by AlRahabi [29] and 50.9% in the study by Haji-Hasani et al. [36] 52.7% in the study by Ilgüy et al. [5], 10% in the study by Lynch and Burke [39], and 27.3% in the study by Mozayeni et al. [38]. The presence of voids indicates incomplete root filling, and adversely affects the treatment prognosis. The presence of voids in the middle and apical thirds of the root canals has a poorer prognosis than voids in the coronal third [41]. The reason for void formation is inadequate access to all parts of the root, or its nonconical shape, preventing the access of condensing instruments to the apical region in lateral compaction and vertical condensation techniques [42].

The frequency of ledge formation was 4.3% in the present study. This value was 6.54% in a study by Zambon da Silva et al. [43] on procedural errors by dental students in Brazil, 2.8% in a study by Vukadinov et al. [44] on procedural errors by dental students in Serbia, 14% in a study by Balto et al. [45] on the performance of dental students in Saudi Arabia, and 17.5% in a study by Dadresanfar et al. [46] on the quality of endodontic treatments by dental students in Tehran, Iran. The prevalence of ledge formation was 24.8% in a study by Eleftheriadis and Lambrianidis [14] on dental students in Greece. This rate was 26% in a study by Mozayeni et al. [38] and 55% in a study by Khabbaz et al. [47]. In some cases, transportation and apical perforation are considered as deep ledges, which can affect the reported frequency rates for ledge formation. The quality of instruction of dental students and the obturation technique may also be responsible for the variations in the reported frequency rates for ledge formation because it has been reported that the passive step-back and balanced force techniques can minimize the risk of ledge formation and transportation [42]. Canal curvature is the main factor responsible for ledge formation and subsequent canal transportation [48]. The incorrect form of the access cavity, faulty detection of the canal path, incorrect working length determination, and not using irrigants are among other contributing factors to ledge formation [42].

In the current study, apical perforation, canal transportation, broken instruments, root fracture, and strip perforation had a frequency of 7.3%, 6.1%, 3%, 1.2%, and 0.6%, respectively. The relatively low frequency of the aforementioned errors was in agreement with the results of other studies in this respect. The frequency of root perforation was 1.1% in the study by Jamani and Fayyad [37]. Apical transportation and perforation had 8.7% and 0.7% frequency rates, respectively, in the study by Mozayeni et al. [38], while Lynch and Burke [39] did not find any evidence of broken instrument or root perforation in their study population. The frequency of broken instruments was 2.5% in the study by Ilgüy et al. [5], while this rate was 9.2% in the study by AlRahabi [29]. The frequency values for apical perforation and canal transportation were both 2.3%. The frequency of broken instruments, missed canals, and apical transportation was 2.8%, 0.3%, and 0.3% in the study by Vukadinov et al. [44]. Apical transportation and root perforation both had a frequency of 7% in the study by Balto et al. [45]. The frequency of root perforation was 2.7% in the study by Eleftheriadis and Lambrianidis [14].

The frequency of strip perforation was very low in the present study. This procedural error may occur due to the use of Gates Glidden drills in the danger zone (root walls close to the furcation area) [14].

In the present study, underfilling and missed canals had a significantly higher frequency in right second molars, and transportation had a significantly higher frequency in right first and second molars. Also, the frequency of root fracture was higher in females than males (*P*=0.025). No other significant differences were noted (*P* > 0.05). Alnowailaty et al. [49] also found that there are more missed canals in females, similar to our findings; however, they found that the frequency of missed canals is highest in the mesiobuccal canals of the first molars. This may be due to the fact that the quality of treatment is largely dependent on the experience and skill of the clinician and the fact that access to the posterior teeth is more difficult in female patients, which may be the cause of more untreated canals.

The use of CBCT for the assessment of procedural errors was a strength of this study due to the high accuracy of this modality for this purpose. De Alencar et al. [50] showed the superior efficacy of CBCT compared with periapical radiography for the detection of endodontic procedural errors.

Further multicenter studies on a larger sample size are required to compare the frequency of procedural errors between general dentists and endodontists. Also, the role of influential factors in the occurrence of procedural errors should be further scrutinized.

## 5. Conclusion

Underfilling, missed canals, and overfilling were the most prevalent procedural errors in mandibular molars in our study population.

## Figures and Tables

**Table 1:** Frequency of underfilling, overfilling, and voids in different canals of endodontically treated mandibular molars.

Procedural error	Canal type	Frequency	Percentage (%)
Underfilling	Distal	41	36.0
	Distobuccal	13	11.4
	Distolingual	14	12.3
	Mesial	5	3.5
	Mesiobuccal	78	68.4
	Mesiolingual	74	64.9

Overfilling	Distal	31	56.4
	Mesial	4	7.3
	Mesiobuccal	21	38.2
	Mesiolingual	22	40.0

Voids	Distal	12	25.5
	Distobuccal	2	4.3
	Distolingual	4	8.5
	Mesial	2	4.3
	Mesiobuccal	20	42.6
	Mesiolingual	27	57.4

**Table 2:** Frequency of missed canals, ledge formation, and apical perforation in different canals of endodontically treated mandibular molars.

Procedural error	Canal type	Frequency	Percentage (%)
Missed canals	Distobuccal	10	17.5
	Distolingual	23	40.4
	Mesial	2	3.5
	Mesiobuccal	14	24.6
	Mesiobuccal 1	2	3.5
	Mesiobuccal 2	4	7.0
	Mesiolingual	12	21.2

Ledge formation	Distal	2	14.3
	Mesiobuccal	6	42.9
	Mesiolingual	8	57.1

Apical perforation	Distal	14	58.3
	Mesial	4	16.7
	Mesiobuccal	8	33.3
	Mesiolingual	6	25.0

**Table 3:** Frequency of canal transportation, root resorption, and apical lesions in different canals of endodontically treated mandibular molars.

Procedural error	Canal	Frequency	Percentage (%)
Canal transportation	Distal	10	50.0
	Distolingual	2	10.0
	Mesiobuccal	8	40.0
	Mesiolingual	5	25.0

Root resorption	Distal	11	61.1
	Mesial	10	55.6
	Mesiobuccal	2	11.1

Apical lesions	Distal	85	56.3
	Mesial	124	82.1
	Mesiobuccal	2	1.3
	Mesiolingual	2	1.3

**Table 4:** Comparison of the frequency of procedural errors in males and females.

Procedural errors	Males	Females	*P* values
Underfilling	55 (30.2%)	59 (40.4%)	0.054
Overfilling	29 (15.9%)	26 (17.8%)	0.652
Voids	27 (14.8%)	20 (13.7%)	0.770
Missed canals	25 (13.7%)	32 (21.9%)	0.052
Ledge formation	10 (5.5%)	4 (2.7%)	0.220
Root fracture	0	4 (2.7%)	0.025
Apical perforation	12 (6.6%)	12 (8.2%)	0.574
Strip perforation	0	2 (1.4%)	0.113
Broken instrument	8 (4.4%)	2 (1.4%)	0.113
Transportation	12 (6.6%)	8 (5.5%)	0.675
Root resorption	13 (7.1%)	5 (3.4%)	0.142
Apical lesion	79 (43.4%)	72 (49.3%)	0.286

**Table 5:** Comparison of the frequency of procedural errors in the right and left mandibular first and second molars.

Procedural errors	Right first molars	Left first molars	Right second molars	Left second molars	*P* values
Underfilling	34 (37.8%)	16 (20.0%)	34 (47.2%)	30 (34.9%)	0.005
Overfilling	15 (16.7%)	11 (13.8%)	13 (18.1%)	16 (18.6%)	0.846
Voids	10 (11.1%)	9 (11.3%)	10 (13.9%)	18 (20.9%)	0.218
Missed canals	12 (13.3%)	10 (12.5%)	22 (30.6%)	13 (15.1%)	0.010
Ledge formation	6 (6.7%)	2 (2.5%)	0	6 (7.0%)	0.085
Root fracture	0	0	2 (2.8%)	2 (2.3%)	0.219
Apical perforation	4 (4.4%)	7 (8.8%)	4 (5.6%)	9 (10.5%)	0.404
Strip perforation	2 (2.2%)	0	0	0	0.150
Broken instrument	0	4 (5.0%)	4 (5.6%)	2 (2.3%)	0.136
Canal transportation	9 (10.0%)	3 (3.8%)	7 (9.7%)	1 (1.2%)	0.037
Root resorption	3 (3.3%)	6 (7.5%)	7 (9.7%)	2 (2.3%)	0.134
Apical lesions	44 (48.9%)	34 (42.5%)	39 (54.2%)	34 (39.5%)	0.253

**Table 6:** Frequency of root canal morphology of mandibular molars according to the Vertucci's classification.

Vertucci's class tooth type	I (%)	II (%)	III (%)	IV (%)	V (%)	VI (%)	VII (%)	VIII (%)	Other (%)	Total (%)
First molar mesial root	2.9	34.6	1.4	55.7	5.4	0	0	0	0	100
First molar distal root	69.4	11.1	4.8	9.1	5.6	0	0	0	0	100
Second molar mesial root	11.6	33.3	4.1	41.4	7.8	0	0	0	1.8	100
Second molar distal root	92.2	1.8	1.8	1.8	0.6	0	0	0	1.8	100

## Data Availability

The data used to support the findings of this study are available from the corresponding author upon reasonable request up to one year after the publication date.
